# Surgical Sympathectomy: Can it be useful in cardiology?

**DOI:** 10.6061/clinics/2020/e1819

**Published:** 2020-04-06

**Authors:** Paulo Manuel Pêgo-Fernandes

**Affiliations:** Departamento de Cardiopneumologia, Instituto do Coracao (InCor), Faculdade de Medicina FMUSP, Universidade de Sao Paulo, Sao Paulo, SP, BR

The first sympathectomy was performed by Alexander in 1889 to treat epilepsy. Over the years, this procedure has been used by countless doctors to treat conditions such as exophthalmos, ischemic lesions due to arterial obstructions, scleroderma, epigastric paralysis, among others 1.

The surgical approach involves resection of the cervical ganglia, inevitably resulting in Horner's Syndrome, which is a combination of signs and symptoms caused by the rupture of a nerve pathway from the brain to the face and eyes, affecting the side of the surgery 2.

In 1916, Thomas Jonnesco performed the first left cardiac sympathetic denervation in a patient with uncontrollable angina and ventricular tachycardia 3. The first endoscopic sympathectomy was performed by Hughes in 1942; however, it was only in the following decade that Kux 4 published his experience with the technique 1. In the 1990s, with the advancement and incorporation of new technologies, video-assisted thoracic sympathectomy became a routine practice, and primary or essential hyperhidrosis became the main indication for thoracic sympathectomy 5.

In video-assisted thoracic sympathectomy, the patient undergoes general anesthesia in a semi-seated position at 45°. The harmonic or electric scalpel and the video camera are inserted through one or two incisions measuring less than one centimeter, and then the sympathetic chain is sectioned at the appropriate levels 6.

The endoscopic approach provides numerous advantages such as reduced postoperative pain, a shorter hospital stay, earlier return to normal activities, and better aesthetic results 7,8.

In cardiology, thoracic sympathectomy has been indicated to treat patients with severe angina pectoris, long QT syndrome 9, and catecholaminergic ventricular tachycardia 10 in selected cases. The most significant effects on cardiac sympathetic activity seem to be related to the excision or blockage of the left stellate ganglion and the subsequent sympathetic chain 11.

A study tested sympathectomy by clipping the lower third of the left stellate ganglion and T3-T4 thoracic nerves using videothoracoscopy in ten patients with class II and III heart failure. The results of this group were compared with those of five patients undergoing clinical treatment in the control group. The results suggest a difference between the two groups regarding improvement in the functional class. The patients undergoing sympathectomy showed a statistically significant improvement in the six-minute walk test and the quality of life questionnaire 12,13.

In patients with cardiac arrhythmia, sympathectomy has been described as a measure to mitigate tachycardia that is refractory to clinical treatment and ablation, especially in cases of channelopathies 14,15.

Another multicenter study evaluated the role of sympathetic cardiac denervation in implantable cardioverter-defibrillator (ICD) patients with structural heart disease and recurrent ventricular tachyarrhythmias after one year. About a third of the patients stopped taking antiarrhythmic drugs with 50% ICD shock-free survival 16.

Sympathetic denervation has been consolidated as a therapeutic option for patients with long QT syndrome in whom drug therapy has already been optimized, and the application of inappropriate cardioverter-defibrillator shocks compromises the quality of life. The effectiveness of sympathectomy was studied in 85 symptomatic patients who had long QT syndrome for six years, with a 45% decrease in cardiac events and an 8% decrease in sudden death 17.

Another study followed up patients who underwent sympathectomy for eight years. Approximately 99% of the patients were symptomatic in the preoperative period, with 48% presenting with aborted cardiorespiratory arrest and 75% presenting with recurrent syncope even with the maximum dose of beta-blockers. After sympathectomy, 46% of patients remained asymptomatic, and there was a 91% decrease in cardiac events and a significant decrease in the length of the QT interval 9.

Thoracic sympathectomy may also be indicated for the treatment of angina in some cases, which was suggested by François Franck based on his studies and understanding of the afferent sympathetic pathways 18.

A T2-T4 sympathetic block causes vasodilation, which can be a useful tool for selected cases of angina refractory to conventional treatment. However, this intervention should be indicated with caution due to hemodynamic instability that can occur if the origin of the angina is not treated 19.

Some cases in the literature describe the effectiveness of sympathectomy in pediatric patients with congenital heart disease and ventricular tachyarrhythmia refractory to surgical treatment 20.

There is a place for surgical sympathectomy in cardiology, which should be considered a therapeutic option for patients with heart failure, long QT syndrome, and catecholaminergic ventricular tachycardia resistant to conventional treatment. A combined treatment of beta-blockers, ablation, and an ICD is the main approach for patients with ventricular arrhythmias.

Currently, the initial phase of a study on whether sympathectomy inhibits the triggering mechanisms of ventricular arrhythmia and reduces the amount of ICD shocks in patients with Chagas cardiomyopathy and recurrent sustained monomorphic ventricular tachycardia has presented very encouraging results.

## References

[1] Hashmonai M (2016). The History of Sympathetic Surgery. Thorac Surg Clin.

[2] Hashmonai M, Cameron AE, Licht PB, Hensman C, Schick CH (2016). Thoracic sympathectomy: a review of current indications. Surg Endosc.

[3] Schwartz PJ, De Ferrari GM, Pugliese L (2017). Cardiac sympathetic denervation 100years later: Jonnesco would have never believed it. Int J Cardiol.

[4] Kux M (1978). Thoracic endoscopic sympathectomy in palmar and axillary hyperhidrosis. Arch Surg.

[5] Wolosker N, Yazbek G, Ishy A, de Campos JR, Kauffman P, Puech-Leão P (2008). Is sympathectomy at T4 level better than at T3 level for treating palmar hyperhidrosis?. J Laparoendosc Adv Surg Tech A.

[6] Wolosker N, Fukuda JM (2015). O tratamento atual da hiperhidrose. J Vasc Bras.

[7] Nicolini EM, Costa VO, Montessi J, Rodrigues GA, Cangussu VV, Reis AFM (2019). Video-assisted thoracic sympathectomy: literature review. Rev Col Bras Cir.

[8] Akil A, Semik M, Fischer S (2019). Efficacy of Miniuniportal Video-Assisted Thoracoscopic Selective Sympathectomy (Ramicotomy) for the Treatment of Severe Palmar and Axillar Hyperhidrosis. Thorac Cardiovasc Surg.

[9] Schwartz PJ, Priori SG, Cerrone M, Spazzolini C, Odero A, Napolitano C (2004). Left cardiac sympathetic denervation in the management of high-risk patients affected by the long-QT syndrome. Circulation.

[10] Collura CA, Johnson JN, Moir C, Ackerman MJ (2009). Left cardiac sympathetic denervation for the treatment of long QT syndrome and catecholaminergic polymorphic ventricular tachycardia using video-assisted thoracic surgery. Heart Rhythm.

[11] Yanowitz F, Preston JB, Abildskov JA (1966). Functional distribution of right and left stellate innervation to the ventricles. Production of neurogenic electrocardiographic changes by unilateral alteration of sympathetic tone. Circ Res.

[12] Pêgo-Fernandes PM, Moreira LF, Souza GE, Bacal F, Bocchi EA, Stolf NA (2010). Endoscopic left sympathetic blockade in the treatment for dilated cardiomyopathy. Arq Bras Cardiol.

[13] Conceição-Souza GE, Pêgo-Fernandes PM, Cruz Fd, Guimarães GV, Bacal F, Vieira ML (2012). Left cardiac sympathetic denervation for treatment of symptomatic systolic heart failure patients: a pilot study. Eur J Heart Fail.

[14] Ajijola OA, Lellouche N, Bourke T, Tung R, Ahn S, Mahajan A (2012). Bilateral cardiac sympathetic denervation for the management of electrical storm. J Am Coll Cardiol.

[15] Wilde AA, Bhuiyan ZA, Crotti L, Facchini M, De Ferrari GM, Paul T (2008). Left cardiac sympathetic denervation for catecholaminergic polymorphic ventricular tachycardia. N Engl J Med.

[16] Vaseghi M, Barwad P, Malavassi Corrales FJ, Tandri H, Mathuria N, Shah R (2017). Cardiac Sympathetic Denervation for Refractory Ventricular [Correction]. J Am Coll Cardiol.

[17] Schwartz PJ, Locati EH, Moss AJ, Crampton RS, Trazzi R, Ruberti U (1991). Left cardiac sympathetic denervation in the therapy of congenital long QT syndrome. A worldwide report. Circulation.

[18] Yoshida K, Inoue T, Hirakawa N, Node K (2008). Endoscopic sympathectomy as a novel strategy for vasospastic angina refractory to medical treatments. J Cardiol.

[19] François-Franck M (1899). Signification physiologique de la résection du sympatique dans la maladie de Basedow, l’épilepsie, l’idiotie et le glaucoma. Bull Acad Méd.

[20] Bonura ED, Moir C, Ackerman MJ, Wackel P (2019). Left cardiac sympathetic denervation for recurrent ventricular tachyarrhythmias in children with congenital heart disease. HeartRhythm Case Rep.

